# The aetiology of severe community-acquired pneumonia requiring intensive care unit admission in the Western Cape Province, South Africa 

**DOI:** 10.7196/AJTCCM.2020.v26i1.035

**Published:** 2020-03-19

**Authors:** A Mazaza, U Lalla, J J Taljaard, T J John, K G John, J Slabbert, C F N Koegelenberg

**Affiliations:** 1 Division of Pulmonology, Faculty of Medicine and Health Sciences, Stellenbosch University, Cape Town, South Africa; 2 Division of Infectious Diseases, Faculty of Medicine and Health Sciences, Stellenbosch University, Cape Town, South Africa; 3 Division of Medical Microbiology, Faculty of Medicine and Health Sciences, Stellenbosch University, Cape Town, South Africa

## Abstract

**Background:**

Community-acquired pneumonia (CAP) is a common condition, with mortality increasing in patients who require intensive care unit (ICU) admission. A better understanding of the current aetiology of severe CAP will aid clinicians in requesting appropriate diagnostic tests and initiating appropriate empiric antimicrobials.

**Objectives:**

To assess the comorbidities, aetiology and mortality associated with severe CAP in a tertiary ICU in Cape Town, South Africa.

**Methods:**

We retrospectively analysed a prospective registry of all adults admitted to the medical intensive care unit at Tygerberg Hospital with severe CAP over a 1-year period.

**Results:**

We identified 74 patients (mean (SD) age 40.0 (15.5) years; 44 females). The patients had a mean (SD) APACHE II score of 21.4 (7.9), and the mean ICU stay was 6.6 days. Of the 74 patients, 16 (21.6%) died in ICU. Non-survivors had a higher mean (SD) APACHE II score than survivors (28.3 (6.8) v. 19.4 (7.1); p<0.001). Mycobacterium tuberculosis (n=16; 21.6%) was the single most common agent identified, followed by *Pseudomonas aeruginosa* (n=9; 12.2%). All *P. aeruginosa* isolates were sensitive to first-line treatment. No organism was identified in 32 patients (43.2%).

**Conclusion:**

*M. tuberculosis* was the single most common agent identified in patients presenting with CAP. The mortality of CAP requiring invasive ventilation was relatively low, with a strong association between mortality and a higher APACHE II score.

## Background

Community-acquired pneumonia (CAP) is one of the leading causes of morbidity and mortality in the developing world.^[1]^ In 2013, the Global Burden of Disease Study^[2]^ found that CAP contributed to 2.7 million deaths worldwide. In South Africa (SA), it is also among the top five causes of natural deaths in adults.^[3]^ CAP accounted for more than 1.7 million total annual hospitalisations in the USA,^[4]^ resulting in a significant economic burden. The 1-month mortality of patients requiring invasive ventilation is 24.4%, increasing to 28.8% in those who develop septic shock.^[5]^ Severe CAP is therefore an absolute indication for admission to the intensive care unit (ICU).^[6]^

There are vast discrepancies between the typical patient presenting with CAP in the developing and the developed world. In the developed world, the incidence of CAP increases with age, and 90% of deaths related to severe pneumonia in the UK occur over the age of 70.^[5]^ In contrast, in sub-Saharan Africa, 55% of deaths occur in those under the age of 70.^[7]^ The reasons for this are multifactorial, with a significant impact from HIV co-infection, high burden of tuberculosis (TB) and vaccine-preventable pathogens such as *Streptococcus pneumoniae* and *Haemophilus influenzae*. ^[8]^ With an estimated HIV prevalence of 12.6% in SA in 2017,^[9]^ those living with HIV contribute greatly to the number of patients requiring hospitalisation for CAP.^[3]^ If HIV is left untreated, it increases the incidence of pneumococcal pneumonia by 17 - 35-fold.^[10]^ A surveillance study conducted in rural Kenya with an HIV incidence of 50 - 75% showed an incidence of pneumococcal pneumonia in 5 and 67 per 1 000 participants in the HIV-negative and HIV-positive populations, respectively.^[7]^

Poor socioeconomic circumstances such as overcrowding, malnutrition and poor indoor ventilation contribute to the burden of tuberculosis in SA, and may also contribute to increased incidence of CAP.^[7]^ Furthermore, it has been shown that 18 to 40% of patients with CAP may test positive for TB, either as a co-existing infection or a primary pathogen.^[11]^

*S. pneumoniae* is the most common identified bacterial pathogen leading to CAP worldwide. Mortality rates as a result of *S. pneumoniae* have been shown to be three times higher than those for other pathogens.^[1]^ Atypical pathogens (*Mycoplasma pneumoniae, Legionella* spp., *Chlamydia pneumoniae*) account for <2% of CAP in SA.^[3]^ Rational antimicrobial prescription is an essential task, as emerging antimicrobial resistance is a global concern.^[3]^ Guidelines for the management of CAP are well documented by internationally reputable sources, but mainly targeted towards the developed world.

Patients with severe CAP treated with combination therapy, including a macrolide, have improved survival compared with those who receive monotherapy.^[5]^ Population-based surveillance programmes must identify the emergence of resistant or atypical organisms in order to guide clinicians on empirical antimicrobial choices for CAP.

The findings of international studies may not always be relevant to SA. We therefore aimed to assess the comorbidities, aetiology and mortality associated with severe CAP in a tertiary ICU in Cape Town, SA.

## Methods

### Study population

All patients admitted to the ICU of Tygerberg Hospital from 1 June 2016 to 31 May 2017 with CAP requiring invasive ventilation were identified from an existing prospectively collected registry. This institution is a 1 380-bed academic hospital in Cape Town, SA. It is one of two academic referral centres in the city, and renders a tertiary service to a population of ~1.5 million, with a local incidence of TB in the order of almost 1 000 per 100 000.^[12]^ The study was approved by the Stellenbosch University Health Research Ethics Committee (ref. no. S18/10/243).

Pneumonia was defined as any patient exhibiting a triad of infection, signs or symptoms localised to the lower respiratory tract and a new radiological infiltrate.^[13]^ We included all patients with complete medical, microbiological and radiological records, and excluded patients hospitalised for >48 hours in our or any other medical care centre.

### Data collection

The demographic data, acute physiology and chronic health evaluation (APACHE) II and comorbidities of all patients were documented, as well as all positive microbiological culture results. Blood cultures and tracheal aspirates were routinely collected from all patients on admission to ICU at the time of the study, and as part of ICU protocol, Xpert MTB/RIF was performed on tracheal aspirates of all patients admitted to the ICU with severe CAP, irrespective of clinical and radiological suspicion for TB. Moreover, urinary testing was performed to detect the presence of *Legionella* antigen, and nasopharyngeal swabs for influenza were performed at the discretion of the treating physician. Final microbiological diagnoses were made in conjunction with the divisions of infectious diseases and microbiology in each case.

### Statistical aspects

Descriptive statistics and χ² or Fisher’s exact tests (where indicated) were performed on dichotomous categorical variables, and *t*-tests on continuous data. Unless stated otherwise, data are displayed as means and standard deviations (SD).

## Results

During the study period, there were 423 admissions to the ICU, with all patients requiring invasive ventilation on admission. We identified 74 patients who met the study criteria (mean (SD) age 40.0 (15.5 years); 44 females). The patients had a mean (SD) APACHE II score of 21.4 (7.9; range 6 - 39), and the mean ICU stay was 6.6 days (range 1 - 41). Of the 74 patients, 16 (21.6%) died in ICU. Major comorbidities included HIV infection (n=19; 25.7%), diabetes mellitus (n=15; 20.3%), chronic lung diseases (n=13; 17.6%) and pregnancy (n=6; 8.1%). Non-survivors had a higher APACHE II score than survivors (28.3 (6.8) v. 19.4 (7.1) (p<0.001)).

In patients who were HIV positive, 2/19 (10.6%) died (odds ratio (OR) 0.34; 95% confidence interval (CI) 0.07 - 1.68; p=0.21), and of those who had CD4 cell counts of under 200 cells/µL, 1/9 died (11.1% (OR 0.42; 95% CI 0.05 - 3.60; p=0.67). Two of 15 patients (13.3%) with diabetes mellitus died (OR 0.49; 95% CI 0.10 - 2.46; p= 0.5) and of those with chronic lung disease, 2/13 died (15.4%; OR 0.61; 95% CI 0.12 - 3.01; p=0.72). Therefore, no comorbidity was protective against the development of severe CAP. There was no mortality observed in pregnant patients. *M. tuberculosis* (n=16; 21.6%) was the single most common organism identified, followed by *Pseudomonas aeruginosa* (n=9; 12.2%). All *P. aeruginosa* isolates were sensitive to first-line treatment (e.g. ceftriaxone). Of the patients with *P. aeruginosa* pneumonia, 22% were HIV-positive and 11% had underlying chronic lung disease. No organism was identified in 32 patients (43.2%). The diagnosis of a single case of *Varicella zoster* pneumonia was based on the clinical picture (including classical skin rash) and chest radiograph findings and there was one case of *Pneumocystis jirovecii*.

## Discussion

We found that *Mycobacterium tuberculosis* was the single most common agent identified, followed by *P. aeruginosa* and *S. pneumoniae*. The ICU mortality was 22%, and the only predictor of mortality was a higher APACHE II score.

There were five confirmed cases of *S. pneumoniae* pneumonia in this study. This may be explained by preceding use of a beta-lactam antibiotic that is routinely commenced at primary and secondary healthcare level, often prior to taking appropriate samples for culture. The low diagnostic yield was also illustrated in international studies in the USA and the Netherlands.^[1,4]^ Despite sophisticated methods used to identify the aetiology of CAP, there was an inability to identify a cause for pneumonia in more than 50% of patients.^[14]^ Another explanation is the indirect reduction in colonisation with *S. pneumoniae* in adults as a result of pneumococcal conjugate vaccination in infants. This herd immunity has been illustrated in international studies,^[15]^ as well as a study conducted in a part of rural SA with a high HIV burden.^[16]^

The present study highlights the significance of TB presenting as lobar consolidation. It is in keeping with previous studies from other high TB-burden countries.^[8,11,17]^ Of the study patients diagnosed with TB, only 15% had HIV co-infection, highlighting the high prevalence of pulmonary TB irrespective of HIV status. Nyamande *et al*. ^[11]^ identified *M. tuberculosis* as the most common organism in a study conducted in KwaZulu-Natal. Among the patients diagnosed with pulmonary TB in the present study, there was a mortality rate of 31% during ICU stay. It should be emphasised that, unlike most blood and tracheal culture techniques, microbiological confirmation of TB remains possible even weeks after empirical therapy. This may therefore have influenced the high yield of *M. tuberculosis* in this study.

Surprisingly, *P. aeruginosa* was identified as an aetiological pathogen in 12% of patients. It is generally considered an uncommon cause of CAP, and mostly associated with nosocomial infections, with associated high mortality and typically extended antibiotic resistance patterns.^[7,18]^ In the context of CAP, it is more likely to be seen in patients who have an identifiable risk factor for developing *P. aeruginosa* pneumonia.^[19]^ *P. aeuruginosa* has been recognised as the second-most common bacterial organism for CAP in patients with HIV co-infection,^[20]^ but this was not reflected in the present study.

The study showed an in-ICU mortality of 22%, even though the average APACHE score predicted a mortality of ~39%. Previous studies conducted in Europe had case-fatality rates ranging from 11% to 48% in ICU.^[1]^ A higher APACHE II score at ICU admission, male sex, chronic heart failure and dialysis were identified as independent risk factors for in-hospital mortality in a study by Li *et al*. ^[21]^

An association between mortality and underlying chronic lung diseases is well described in the literature. Vidal *et al*.^[22]^ identified a 23% mortality in patients with CAP and comorbid chronic respiratory disease in a study conducted in Portugal. A retrospective cohort study in the USA showed a statistically significant increase in 30-day mortality, rate of ICU admission and length of hospital stay among patients with co-existing COPD who were hospitalised for CAP.^[23]^ In our population, however, mortality was not associated with underlying lung diseases.

## Study limitations

Our study had some limitations. Owing to the small sample size, we were not able to identify statistically significant associations between certain risk factors and mortality. No organism was identified in just over 40% of cases, many of whom in all probability may have had *Streptococcus pneumoniae* or other common causes of bacterial pneumonia. Testing for viral infections and atypical pathogens (including *Legionellae*) was only performed when requested and not routinely, which may have led to a significant proportion of cases not being accurately diagnosed.

## Conclusion

*M. tuberculosis* was the single-most common agent identified in patients presenting with severe CAP requiring ventilation. The relatively high percentage of confirmed community-acquired *P. aeruginosa* could be related to the concomitant severe drought experienced at the time of the study. The mortality of CAP requiring invasive ventilation was relatively low, and the only predictor of mortality was a higher APACHE II score.
